# Is Vivisection Immoral?

**Published:** 1892-07-09

**Authors:** Ernest Bell

**Affiliations:** Thurlow Road, Hampstead


### Editor's Letter-Box.

[Our correspondents are reminded that prolixity is a great bar to publication, and that brevity of style and conciseness of statement greatly facilitate early insertion.]

### IS VIVISECTION IMMORAL?

Sir,—I hope you will allow me, as an anti-vivisectionist, to make a few remarks on the article in your last issue, which seems to me to fail, logically, from the writer having fallen into the not uncommon error of treating "nature" as though she was a veritable dame, responsible for her actions, instead of merely a convenient term denoting certain facts and conditions. The gist of your argument, if I rightly understand it, is that "nature makes her own exactions at the hands of the unwilling as well as of the willing," and, therefore, the vivisector has a right to do the same. If now the physiologist is to be regarded as an irresponsible part of nature, like the flash of lightning or the poisonous malaria, or even like the beast of prey, with freedom of action but no sense of responsibility, then his acts might be justifiable morally, if such a term could be applied to an irresponsible agent. But even then it would be a natural impulse in us to limit the evil he does as much as possible as in the other cases instanced. If, on the other hand, he is a responsible being, as we all hold him to be, with knowledge of right and wrong, then in claiming the right to dispense evil he is assuming the part, not of nature, but of the God of nature, and that with regard to the very attribute which is most solemn and incomprehensible to us. For is it not just the presence in nature of this vicarious suffering of the innocent, so mysterious to us, so unjust, according to our sense of justice, which has first led so many souls to doubt the existence of a beneficent God? We rebel against it in nature, and suffer it only because we are powerless to alter it, humbly hoping that we may some day find some explanation of the seeming wrong. If vivisection can be defended only by the plea that it is in keeping with the "curse" which lies upon the world, then surely it is sufficiently condemned.—I am, &c.,

Thurlow Road, Hampstead. Ernest Bell, M.A.

[Vivisection is not defended on the ground that it is in keeping with the "curse." What is the "curse" but a doubtfully existent fact for whose shadowy substance a theological case was long ago manufactured? The writer of the article strove to show that vivisection is not unaccordant with the "constitution and course of nature," and, therefore, not "irreligious, immoral, or inhumane"—Ed.]

